# Osteopathic versus allopathic medical school pathology curricula: a survey of medical students at Michigan State University

**DOI:** 10.1016/j.acpath.2025.100164

**Published:** 2025-02-18

**Authors:** Curtiss Johnson, Meredith Herman, Basma Klump, Christina Silva, Jessica Maupin, Casey P. Schukow, Justin Tran, Paul Kowalski

**Affiliations:** a Michigan State University College of Osteopathic Medicine, East Lansing, MI, USA; b University of Michigan, Department of Pathology, Ann Arbor, MI, USA; c The Ohio State University, Department of Pathology, Columbus, OH, USA; d Corewell Health William Beaumont University Hospital, Department of Pathology, Royal Oak, MI, USA; e Michigan State University College of Human Medicine, Grand Rapids, MI, USA

**Keywords:** Allopathic, Osteopathic, Pathology education, Undergraduate medical education

## Abstract

Despite guidelines for pathology undergraduate medical education set forth by the Association of American Medical Colleges, American Medical Association, Liaison Committee on Medical Education, and Commission on Osteopathic College Accreditation, there is sparse literature regarding differences in pathology curricula between allopathic and osteopathic institutions. As programs alter curricula to adapt to the ever-increasing breadth and depth of medical knowledge, there is concern for lost educational opportunities in pathology and a growing need for research on the landscape of pathology undergraduate medical education in medical schools nationwide. An Institutional Review Board approved, voluntary 22-item survey regarding pathology curricula was distributed to allopathic and osteopathic medical school students at Michigan State University from July 2022 to January 2023. The total number of responses was 363 (n = 363; 22.6% allopathic, 77.4% osteopathic). We present data on pathology education at a university that features both an allopathic and osteopathic college of medicine while focusing on factors that influence medical students’ perceptions of pathology. Statistically significant differences (*P*≤0.05) in responses—favoring Michigan State University osteopathic students over their allopathic counterparts—were observed in several areas: the perception of pathology as a medical versus surgical specialty (*P* 0.014), acknowledgement of a dedicated pathology course (*P* 0.002), and awareness of pathology-specific content (*P* < 0.001). Allopathic students expressed a greater desire for pathology exposure (*P* 0.003). This study highlights the variable exposure of pathology between two different curriculums and suggests that, while traditionally primary-care-focused, osteopathic medical programs may offer stronger pathology education and exposure.

## Introduction

Medical student competency in pathology is a core principle for medical education and clinical practice for Liaison Committee on Medical Education (LCME) accreditation of allopathic programs. These standards are set forth by the American Medical Association and the Association of American Medical Colleges, which is recognized by the U.S. Department of Education.^1^ With respect to curriculum content, the LCME expects “the faculty of a medical school ensure that the medical curriculum provides content of sufficient breadth and depth to prepare medical students for entry into any residency program and for the subsequent contemporary practice of medicine,” (Standard 7).^2^ Furthermore, the Association for Academic Pathology (formerly the Association of Pathology Chairs) has created working groups to shape pathology content and curriculum at LCME-accredited medical schools, but there is no mention of its involvement at Commission on Osteopathic College Accreditation (COCA)-accredited osteopathic medical schools.^3^

Similar competencies for pathology exist across 66 accredited osteopathic medical schools, as set forth by the COCA.^4^ These guidelines are enforced by the American Association of Colleges of Osteopathic Medicine (AACOM).^5^ As per the AACOM core competency report, osteopathic medical students are assessed for osteopathic principles and practices, which states that students must use the relationship between structure and function to promote a core osteopathic tenant and biologic principle. A subcategory further states that students must learn to “apply knowledge of the biomedical sciences, such as functional anatomy, physiology, biochemistry, histology, pathology, and pharmacology, to support the appropriate application of osteopathic principles.”^5^ The AACOM website lists a sample curriculum that suggests pathology be a first-year core curriculum topic; however, it is not mentioned in the clinical clerkship curriculum.^5^

While it is a core component of pre-clerkship education, according to the LCME Annual Medical School Questionnaire Part II, pathology needs not be a required clerkship discipline.^6^ Rotation requirements vary across allopathic medical schools. However, the recommended rotations (many of which are required) include ambulatory care, anesthesiology, critical care, emergency medicine, internal medicine, neurology, obstetrics and gynecology, pediatrics, psychiatry, radiology, general surgery, and surgical specialties.^7^ Similarly, pathology is not explicitly included in the recommended clerkship curriculum as per the AACOM; the required clerkship rotations for osteopathic medical students are family medicine, internal medicine, general surgery, pediatrics, psychiatry, obstetrics and gynecology, and emergency medicine.^5^ This raises the question of why pathology is recognized as a core discipline but not as a core specialty in clinical education. If the major accrediting organizations do not offer guidance and recommendations on incorporating pathology into the clinical training of medical students, pathology will likely continue to be excluded from clinical experience among medical schools.

Despite published curricular guidelines, there are limited studies on how the pathology curriculum is implemented and delivered across allopathic and osteopathic undergraduate medical education (UME) institutions.^8^ Michigan State University (MSU) houses two medical colleges: the allopathic medical school, the MSU College of Human Medicine (MSUCHM) and the osteopathic medical school, the MSU College of Osteopathic Medicine (MSUCOM). The MSUCHM has a student body of approximately 750 students, admitting around 190 students a year.^9^ The MSUCOM has a student body of approximately 1200 students, admitting 300 students a year.^10^ Neither MSUCHM nor MSUCOM has a dedicated pathology department. There are few pathology faculty at both programs, most of whom are employed through the MSU Department of Physiology.^8^^,^^11^ Both programs offer a 2-year pre-clerkship curriculum. The MSUCHM presents pathology in the first year through a 4-week histology and pathology course, followed by independent study via a shared-discovery curriculum in which students acquire knowledge and demonstrate understanding through a mix of “flipped-classroom” sessions, case-based learning, and small-group discussion.^12^ The MSUCOM introduces pathology in year one as part of a foundational biomedical science course, with additional content integrated into organ systems courses.^12^^,^^13^ Neither program mandates a pathology rotation during clerkship (years 3 and 4), but both offer it as an elective at an institution chosen by the student.^12^^,^^14^

Considering the similar accreditation standards for allopathic and osteopathic medical schools, yet the different curricula, we sought to elucidate the distinctions in medical student experiences in pathology across the two medical schools. Additionally, we revealed how these curricular differences may shape medical students' perceptions of pathology and their inclination to specialize in this field.

## Materials and methods

This was an IRB-approved (MSU IRB protocol number: STUDY00007931), qualitative cross-sectional study in which an electronic 22-item Qualtrics survey regarding respective pathology curricula was administered to all enrolled allopathic and osteopathic students over a seven-month period from July 2022 to January 2023. Surveys were distributed via email through each school's administration, twice to the MSUCHM (allopathic) and three times to the MSUCOM (osteopathic), and social media accounts including Facebook and Twitter. The survey was completely anonymous and voluntary, and students' participation in the survey did not impact their course standings or requirements. No medical records or personal identifying information was used or collected in the survey, and this study was administered separately from any pre-clerkship or clerkship course. A copy of the survey questions is included for reference (Supplementary Material 1). Comparisons on nominal variables were tested using a chi-squared test for independence, whereas comparisons on ordinal variables were made using a chi-squared test for trend. All analyses were conducted using R statistical software (version 4.2.2; R Core Team, 2022) and GraphPad Prism (version 10.2.3). The coin package was used to run chi-squared tests for Table 1 (version 1.4–2; Hothorn, Hornik, van de Wiel, & Zeileis, 2006). GraphPad Prism (version 10.2.3) was used to make the graphs and analyze chi-squared tests for all figures.

**Table 1:** Survey results: The results of 19 questions from the survey that was distributed to the College of Osteopathic Medicine (COM) and College of Human Medicine (CHM) at Michigan State University. *P*-values <0.05 are considered statistically significant differences in results between COM and CHM students (highlighted by asterisk).

	COM (N = 281)	CHM (N = 82)	*P*-value
**1. Does your school have a dedicated pathology course in pre-clerkship?**			0.002∗
No	73 (26.0%)	34 (41.5%)	
Yes	132 (47.0%)	21 (25.6%)	
Unsure	48 (17.1%)	18 (22.0%)	
NA	28 (10.0%)	9 (11.0%)	
**2. Does your school offer a required pathology clerkship rotation?**			0.017∗
No	104 (37.0%)	42 (51.2%)	
Yes	5 (1.8%)	3 3.7%)	
Unsure	144 (51.2%)	28 (34.1%)	
NA	28 (10.0%)	9 (11.0%)	
**3. Do you consider pathology more so a surgically- or medically-based specialty?**			0.014∗
Equally surgically and medically based	118 (42.0%)	21 (25.6%)	
Medically based	127 (45.2%)	48 (58.5%)	
Surgically based	6 (2.1%)	4 (4.9%)	
NA	30 (10.7%)	9 (11.0%)	
**4. Have you ever interacted with a pathologist at your medical school or in an extracurricular setting?**			0.180
No	77 (27.4%)	16 (19.5%)	
Yes	162 (57.7%)	55 (67.1%)	
Unsure	14 (5.0%)	2 (2.4%)	
NA	28 (10.0%)	9 (11.0%)	
**5. How many different pathologists have taught you?**			0.536
0–1	120 (42.7%)	36 (43.9%)	
2–3	88 (31.3%)	29 (35.4%)	
4–6	16 (5.7%)	2 (2.4%)	
7+	1 (0.4%)	0 (0%)	
NA	56 (19.9%)	15 (18.3%)	
**6. Is pathology regularly integrated during other systems courses throughout your pre-clerkship education?**			0.039∗
No	7 (2.5%)	7 (8.5%)	
Yes	188 (66.9%)	53 (64.6%)	
Unsure	32 (11.4%)	7 (8.5%)	
NA	54 (19.2%)	15 (18.3%)	
**7. Did you know that pathologists went to medical school?**			0.520
No	19 (6.8%)	4 (4.9%)	
Yes	208 (74.0%)	63 (76.8%)	
NA	54 (19.2%)	15 (18.3%)	
**8. Have you observed the clinical work of a pathologist at any point during your medical experience?**			0.287
No	169 (60.1%)	56 (68.3%)	
Yes	46 (16.4%)	10 (12.2%)	
Unsure	10 (3.6%)	1 (1.2%)	
NA	56 (19.9%)	15 (18.3%)	
**9. I am interested in pursuing pathology as a residency.**			0.232
Definitely not	91 (32.4%)	32 (39.0%)	
Probably not	85 (30.2%)	28 (34.1%)	
Might or might not	40 (14.2%)	7 (8.5%)	
Probably yes	6 (2.1%)	0 (0%)	
Definitely yes	4 (1.4%)	0 (0%)	
NA	55 (19.6%)	15 (18.3%)	
**10. How much exposure to pathology have you had at your school?**			0.123
None at all	20 (7.1%)	5 (6.1%)	
A little	52 (18.5%)	20 (24.4%)	
A moderate amount	102 (36.3%)	34 (41.5%)	
A lot	39 (13.9%)	6 (7.3%)	
A great deal	12 (4.3%)	1 (1.2%)	
NA	56 (19.9%)	16 (19.5%)	
**11. I believe my formal pathology exposure in medical school should be…**			0.003∗
Much less	5 (1.8%)	0 (0%)	
Somewhat less	13 (4.6%)	1 (1.2%)	
About the same	129 (45.9%)	30 (36.6%)	
Somewhat more	68 (24.2%)	31 (37.8%)	
Much more	9 (3.2%)	4 (4.9%)	
NA	57 (20.3%)	16 (19.5%)	
**12. How satisfied are you with the quality of instruction related to pathology at your medical school?**			0.128
Extremely dissatisfied	6 (2.1%)	2 (2.4%)	
Somewhat dissatisfied	17 (6.0%)	9 (11.0%)	
Neither satisfied nor dissatisfied	50 (17.8%)	12 (14.6%)	
Somewhat satisfied	67 (23.8%)	30 (36.6%)	
Extremely satisfied	76 (27.0%)	13 (15.9%)	
NA	65 (23.1%)	16 (19.5%)	
**13. What level of importance would you rate the work of a pathologist in patient care in comparison to other specialties (e.g., family medicine, surgery, etc.)**			0.974
Not at all important	2 (0.7%)	0 (0%)	
Slightly important	11 (3.9%)	4 (4.9%)	
Moderately important	48 (17.1%)	15 (18.3%)	
Very important	109 (38.8 %)	33 (40.2%)	
Extremely important	49 (17.4%)	15 (18.3%)	
NA	62 (22.1%)	15 (18.3%)	
**14. Pathology exposure is equivalent to most other biomedical science content (e.g. pharmacology, physiology, anatomy, etc.) at my medical school.**			0.279
Strongly disagree	10 (3.6%)	4 (4.9%)	
Somewhat disagree	39 (13.9%)	14 (17.1%)	
Neither agree nor disagree	47 (16.7%)	14 (17.1%)	
Somewhat agree	71 (25.3%)	26 (31.7%)	
Strongly agree	50 (17.8%)	9 (11.0%)	
NA	64 (22.8%)	15 (18.3%)	
**15. My medical school curriculum makes obvious which content being taught is pathology related.**			<0.001∗
Strongly disagree	10 (3.6%)	2 (2.4%)	
Somewhat disagree	14 (5.0%)	13 (15.9%)	
Neither agree nor disagree	33 (11.7%)	15 (18.3%)	
Somewhat agree	90 (32.0%)	23 (28.0%)	
Strongly agree	69 (24.6%)	13 (15.9%)	
NA	65 (23.1%)	16 (19.5%)	
**16. Pathology content is a priority of my medical school curriculum.**			0.418
Strongly disagree	5 (1.8%)	2 (2.4%)	
Somewhat disagree	26 (9.3%)	10 (12.2%)	
Neither agree nor disagree	60 (21.4%)	15 (18.3%)	
Somewhat agree	80 (28.5%)	32 (39.0 %)	
Strongly agree	43 (15.3%)	7 (8.5%)	
NA	67 (23.8%)	16 (19.5%)	
**17. I have a strong general interest in learning pathology and pathophysiology.**			0.679
Neither agree nor disagree	50 (17.8%)	9 (11.0%)	
Somewhat agree	82 (29.2%)	33 (40.2%)	
Somewhat disagree	32 (11.4%)	11 (13.4%)	
Strongly agree	42 (14.9 %)	12 (14.6%)	
Strongly disagree	11 (3.9%)	2 (2.4%)	
NA	64 (22.8%)	15 (18.3%)	
**18. How well do you feel your program has prepared you in understanding pathologists' role in the healthcare team?**			0.672
Not well at all	25 (8.9%)	9 (11.0%)	
Slightly well	49 (17.4%)	16 (19.5%)	
Moderately well	78 (27.8%)	22 (26.8%)	
Very well	48 (17.1%)	16 (19.5%)	
Extremely well	13 (4.6%)	3 (3.7%)	
NA	68 (24.2%)	16 (19.5%)	
**19. I am comfortable with pathology questions on board exams.**			0.281
Extremely uncomfortable	28 (10.0%)	5 (6.1%)	
Somewhat uncomfortable	46 (16.4%)	15 (18.3%)	
Neither comfortable nor uncomfortable	54 (19.2%)	15 (18.3%)	
Somewhat comfortable	74 (26.3%)	29 (35.4%)	
Extremely comfortable	9 (3.2%)	2 (2.4%)	
NA	70 (24.9%)	16 (19.5%)	

## Results

The total number of responses between both student bodies was 363 (n = 363). There were 82 responses from allopathic students, (about 11% of the student body, 22.6% of the survey responses) and 281 responses from osteopathic students (about 23% of the student body, 77.4% of the survey responses). Most respondents from both colleges (230 respondents [63.4%]) indicated that they were in their pre-clinical years (1st- and 2nd-year medical students) at the time of data collection. Full results for the remaining survey items are shown in Table 1.

**Fig. 1:**
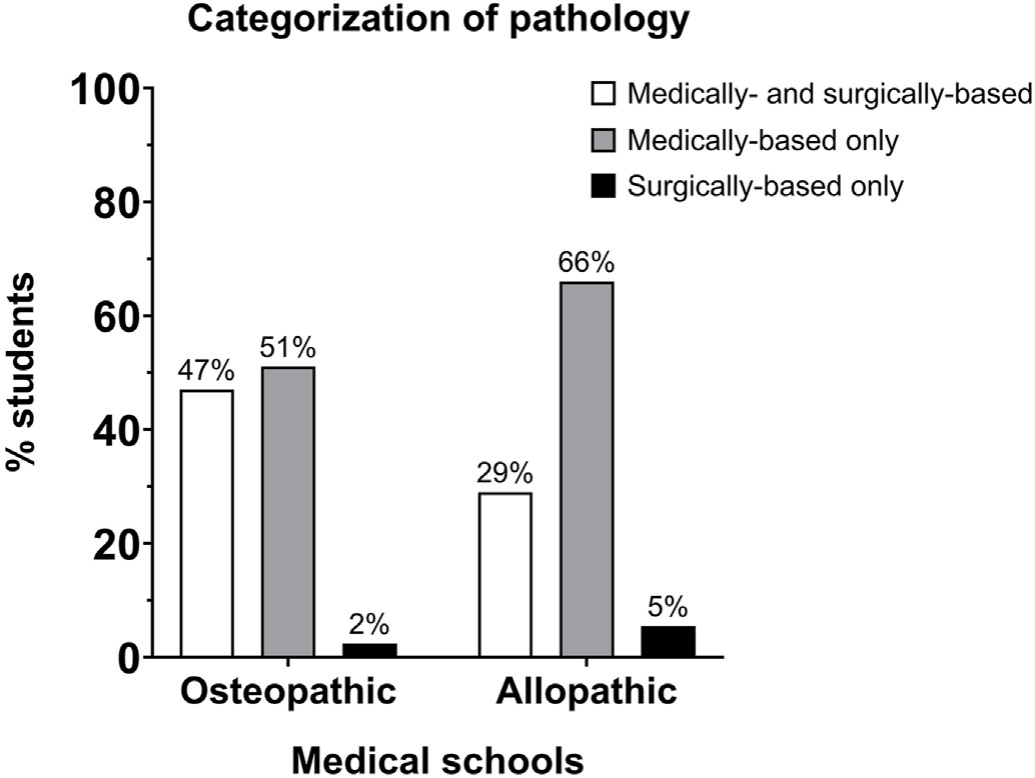
Categorization of pathology: the distribution of the number of osteopathic medical students who categorize pathology as either a medical or a surgical specialty was different from the distribution of allopathic medical students. χ^2^ = 8.541, df = 2, *P*=0.0140.

**Fig. 2:**
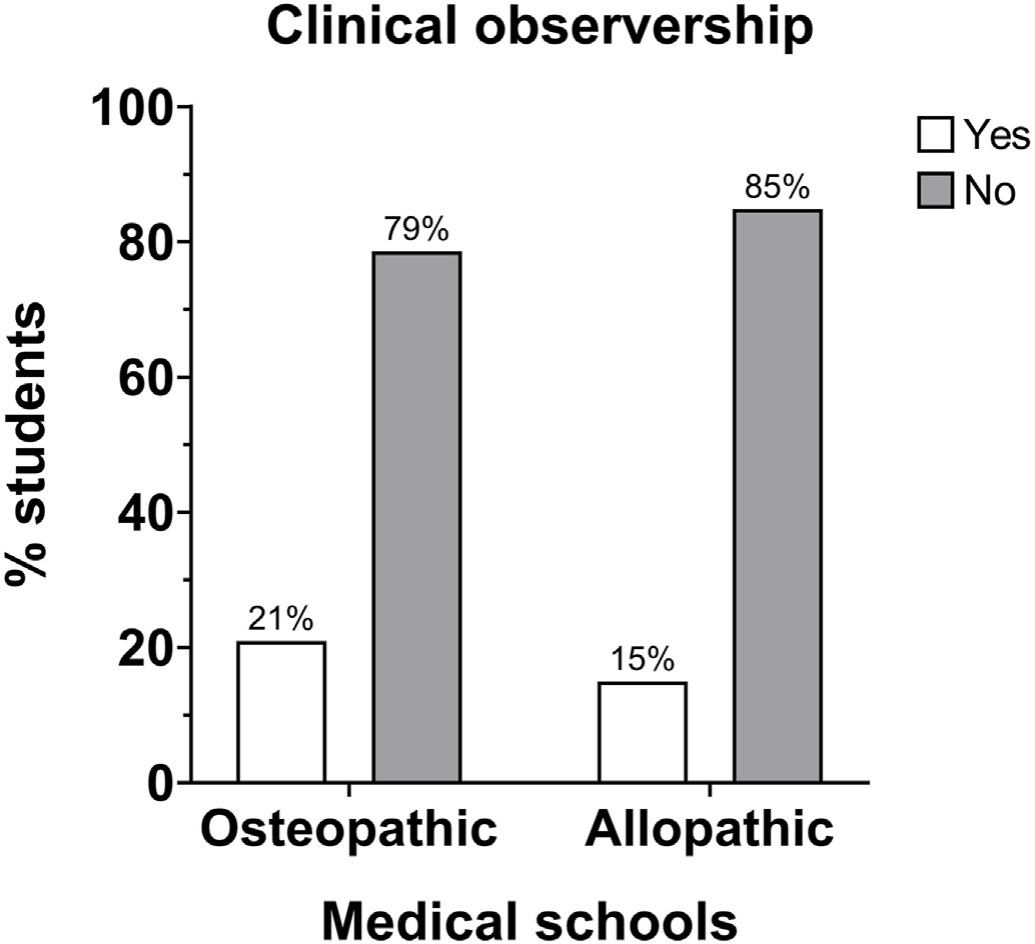
Clinical observership: the distribution of the number of students participating in pathology-related clinical observership in osteopathic medical school was consistent with that of allopathic medical school. χ^2^ = 1.234, df = 1, *P* = 0.2667.

Approximately 67% of osteopathic students and 70% of allopathic students did not believe that their program educated them well in understanding the role of pathologists in patient care (Table 1 Question #18). Between 74% and 77% of students understood that pathologists complete medical school training (Table 1 Question #7). However, only 42% of osteopathic students and significantly fewer allopathic students (26%) considered pathology as an equally surgery- and medicine-based specialty (Fig. 1, Table 1 Question #3). Furthermore, 79–85% of students have not observed the work of pathologists (Fig. 2).

**Fig. 3:**
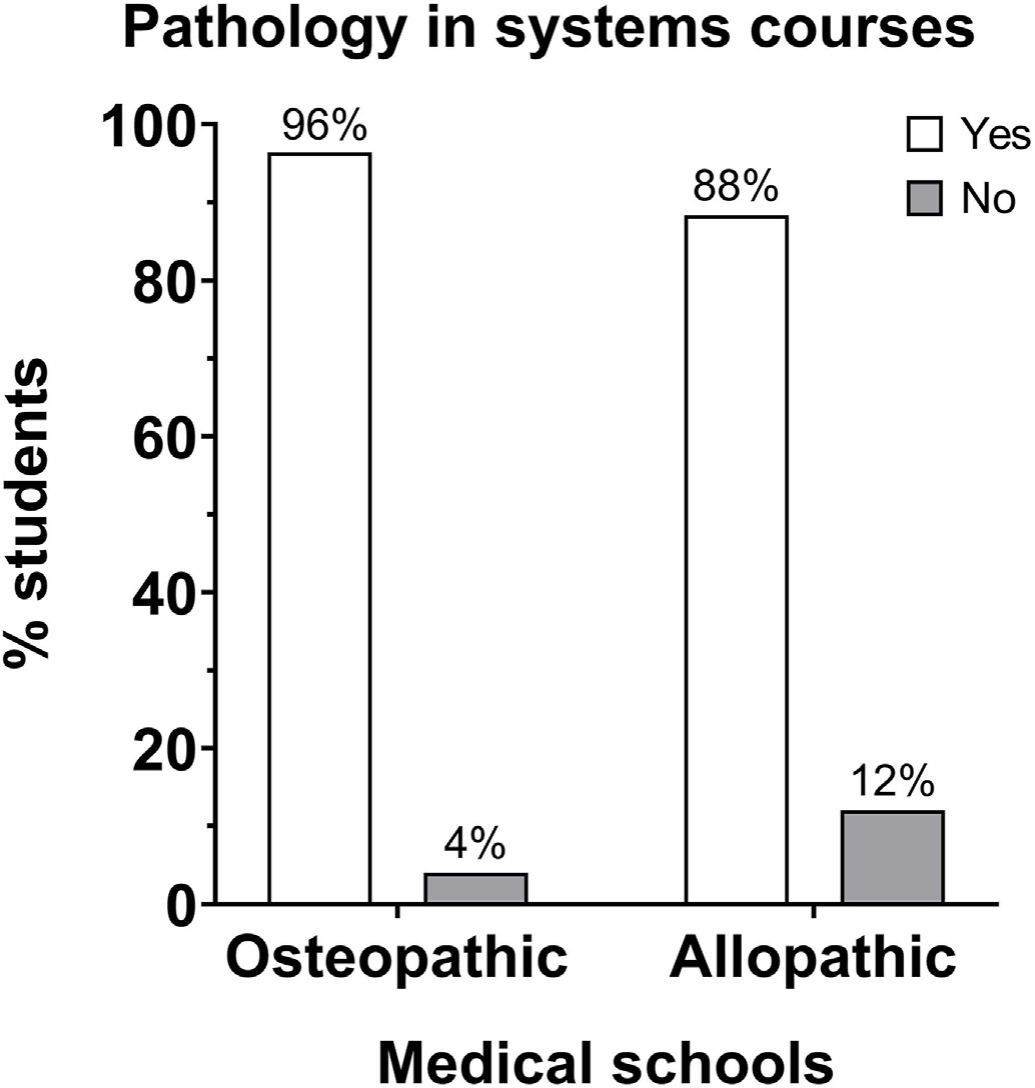
Pathology in systems courses: the distribution of the number of osteopathic medical students who think that pathology is integrated into systems courses was different from the distribution of allopathic medical students. χ^2^ = 5.769, df = 1, *P* = 0.0163.

**Fig. 4:**
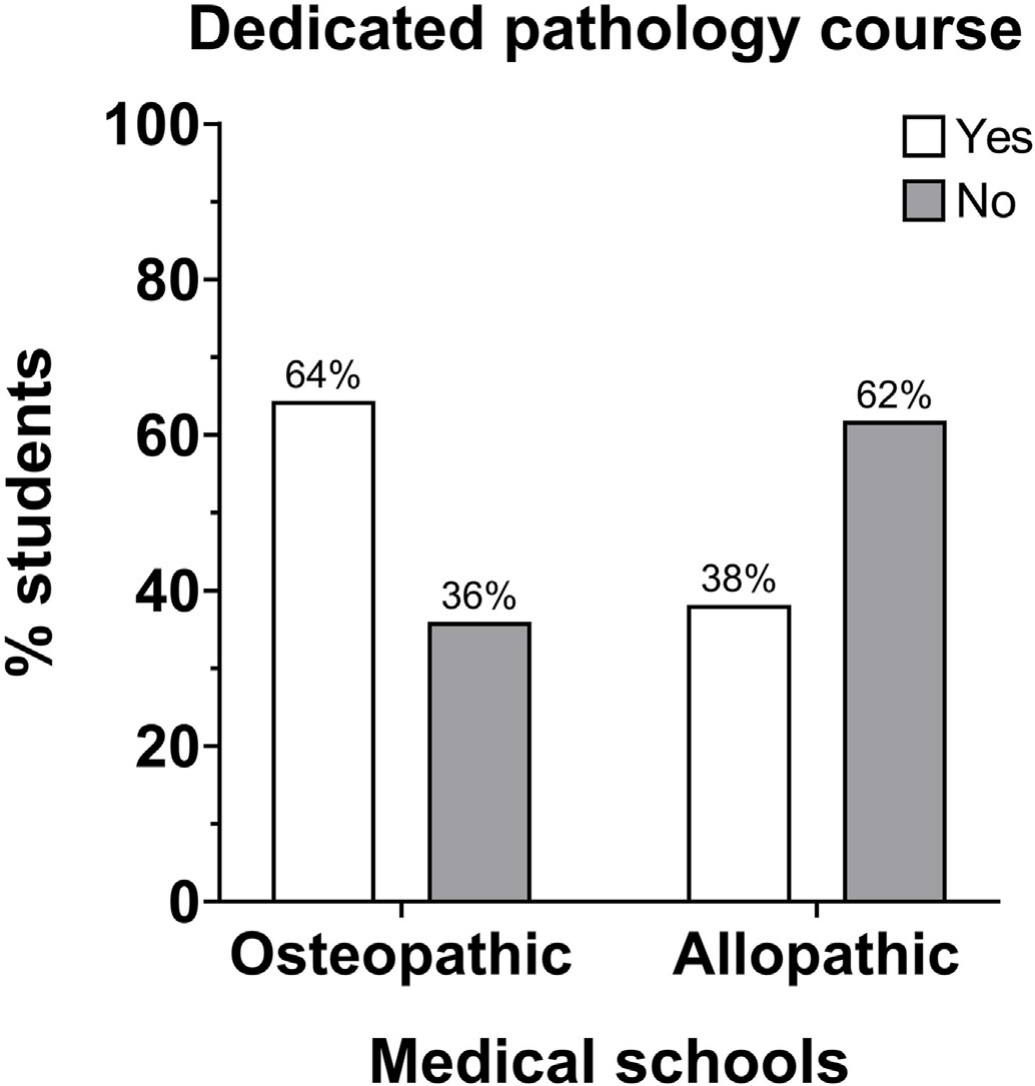
Dedicated pathology course: the distribution of the number of students who admit to having a dedicated pathology course in pre-clerkship in osteopathic medical school was different from the distribution of allopathic medical students. χ^2^ = 12.3, df = 1, *P* = 0.0005.

Markedly higher numbers of osteopathic (96%) and allopathic (88%) medical students reported exposure to pathology-related content incorporated in their schools’ systems courses than students who reported no pathology incorporation in their courses (Fig. 3). Forty-seven percent of osteopathic students reported a dedicated pathology course in pre-clerkship, whereas interestingly, a significantly lower number of allopathic students (21%) reported a dedicated pathology course during pre-clerkship at their school (Fig. 4).

**Fig. 5:**
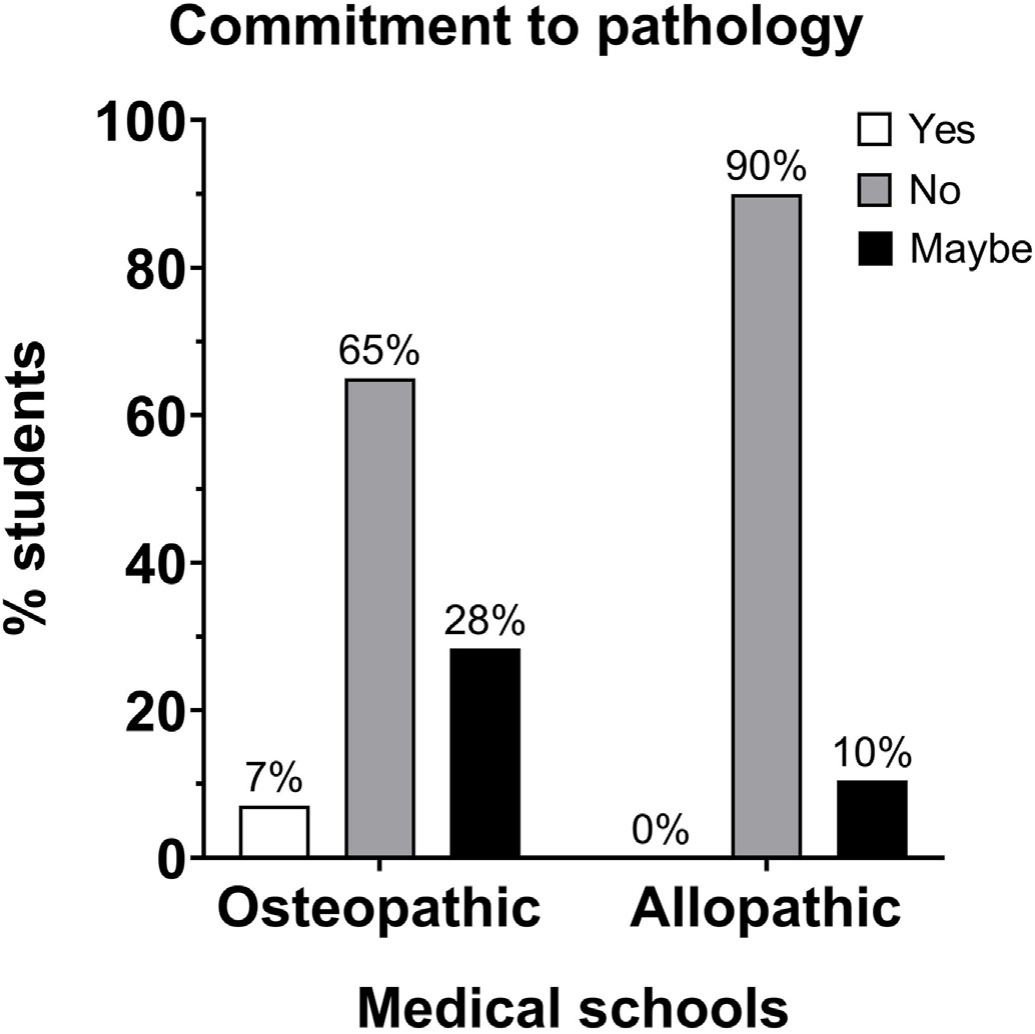
Commitment to pathology: the distribution of the number of osteopathic medical students who state their commitment to pathology was different from the distribution of allopathic medical students. χ^2^ = 15.12, df = 2, *P* = 0.0005.

Less than 4% of allopathic and osteopathic students reported a required pathology clerkship rotation, and less than 16% reported participation in observership or clinical enrichment in the field of pathology (Fig. 2, Table 1 Question #2, #8). Therefore, despite incorporating pathology-related content in pre-clerkship, students' exposure to the field is minimal and does not foster a future commitment to pursuing pathology in the future (Fig. 5, Table 1 Question #2).

Limited exposure to pathology-related course content and activities during medical school predisposes individuals—evidently more in allopathic students than osteopathic students—to a lack of awareness in pathology (Table 1 and Fig. 1, Fig. 2, Fig. 3, Fig. 4). A deficiency in information and continuous lack of exposure to the discipline may dissuade students from pursuing a career in pathology. Evidently, 7% of osteopathic survey participants and 0% of allopathic students expressed a commitment to the field of pathology (Fig. 5).

**Fig. 6:**
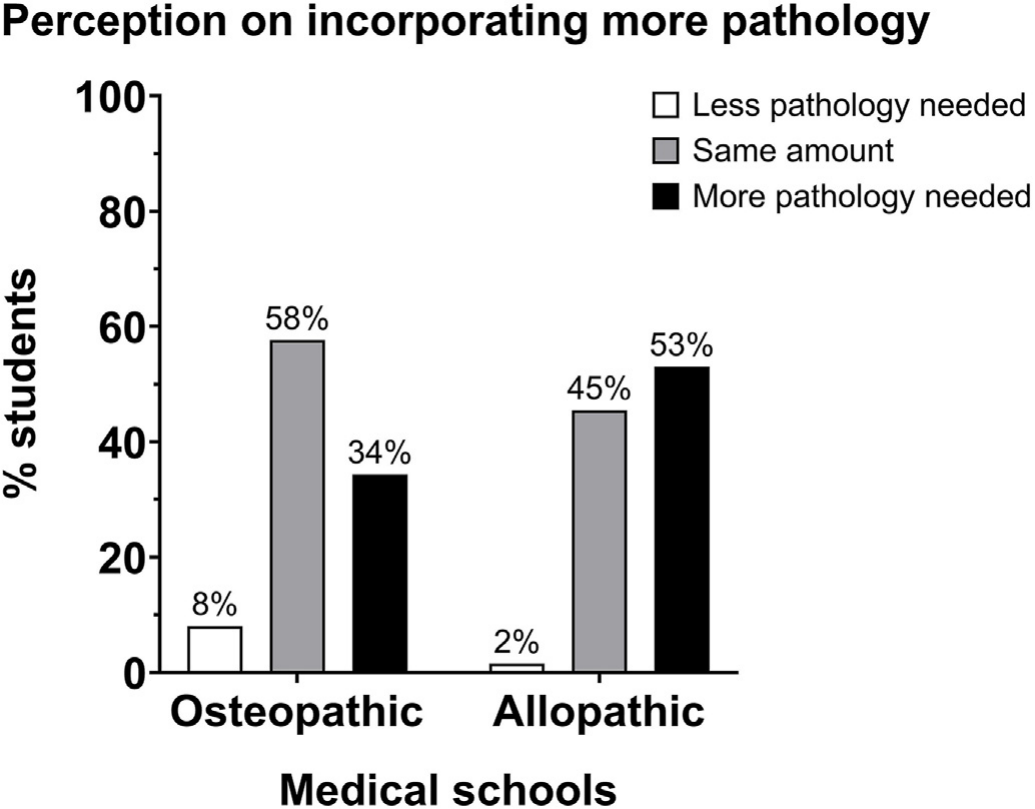
Perceptions on incorporating more pathology: the distribution of the number of osteopathic medical students on the need for more pathology training during medical school was different from the distribution of allopathic medical students. χ^2^ = 9.271, df = 2, *P* = 0.0097.

A plurality of the students surveyed reported an interest in learning pathology- and pathophysiology-related subjects (Table 1 Question #17). In fact, 53% of allopathic students, in comparison with 34% osteopathic students, believe their medical school curriculum needed to incorporate more pathology (Fig. 6). Additionally, pathology exposure outside of the curriculum is deficient, as over 80% of students have not been exposed to the work of pathologists (Fig. 2).

Despite interest in pathology content by both allopathic and osteopathic students (Table 1 Question #17), the vast majority of students had no interest in pathology as a career (Fig. 5). While both allopathic and osteopathic students in this study expressed an overall favorable awareness and attitude toward pathologists, a subset of respondents expressed that they were not aware that pathologists completed medical school.

One of the most notable findings was the difference in opinions between osteopathic and allopathic students regarding their respective colleges' integration and presentation of pathology curricula. Osteopathic students were more likely to recognize and value the pre-clerkship courses and lecture content specifically dedicated to pathology. In contrast, allopathic students expressed a desire for a formal integration of pathology into their medical school curriculum (Fig. 6).

## Discussion

This study draws a distinct comparison of perceptions and curriculum within a single university, underscoring the need for further studies across additional U.S. allopathic and osteopathic medical schools.

One similarity between the two separate medical schools is that there is no affiliated university tertiary medical center. Therefore, clinical education is predominantly community based. The variability in pathology exposure across numerous community hospitals results in a lack of standardized pathology education. Consequently, most respondents reported never having observed the clinical work of a pathologist (Fig. 2). This is likely consistent with other medical schools nationwide, as most UME curricula do not require a pathology clerkship for graduation.^5^^,^^6^

This raises the question of how to engage more medical students in pathology, both within and beyond their school's curriculum. In addition to institutional curricular revisions, strategies such as “a day in the lab” and postsophomore fellowships have been studied to enhance knowledge of laboratory medicine and recruit medical students to the specialty.^15^^,^^16^ However, despite efforts to address current gaps in UME, these initiatives have shown limited success in significantly increasing the number of U.S. medical students applying for and matching into pathology residency programs.^17^ Recent efforts have focused on delivering pathology content through virtual platforms, enhancing accessibility and facilitating learning for U.S. medical students.^18^, ^19^, ^20^ Irrespective of the approach used, there is an evident need for exposure to pathology during both pre-clerkship and clerkship training and awareness of the importance of laboratory medicine and the role of pathologists in patient care.

One unique aspect to our focus institution is that the medical schools share a student-led pathology interest group. While there is limited research on the role of pathology interest groups in shaping students’ interests in pathology, there is evidence that they may fill a gap in the curriculum by providing awareness of resources, mentorship, and valuable in-person pathology experiences.^21^, ^22^, ^23^ Increasing exposure is essential to dispel common myths and misconceptions about pathology and laboratory medicine. For many medical students, their initial encounter with the field occurs through mandatory histology labs in organ system courses.^24^ The abstract nature of pathology, combined with a common aversion to histology during the pre-clerkship phase, can lead to a lasting unfamiliarity with the subject and discourage students from exploring the field further.^25^ A study by Holland and Bosch revealed that even a dedicated pre-clerkship pathology course does not significantly alter medical students' perceptions of pathology. A potential way to improve overall perceptions toward the specialty is for pathologists to actively promote the specialty during instructional sessions.^26^ Addressing and revising obstacles that shape medical students' perceptions of pathology could lead to a systemic change, ultimately attracting learners from various specialties and potentially recruiting future pathologists to the field of pathology.

Medical school faculty members also play a significant role in shaping students' specialty interests, a sentiment that is shared among both allopathic and osteopathic residency applicants.^17^^,^^27^ Outside of their clinical instruction duties, faculty members (particularly in pathology) are a source of mentorship and encouragement for students willing to explore the field.^25^^,^^28^ It is not surprising that pathologist representation in medical school curriculum committees and executive positions (or lack thereof) can influence students’ awareness and interest in pathology, which more often than not is a deterrent for students to pursue it.^24^^,^^28^ Encouragingly, most students in our study reported interactions with at least one pathologist during medical school. Including pathologists in administrative roles, such as appointed deans, may enhance pathology education and foster sustainable curricular initiatives to increase student exposure to the field earlier in their training.^29^ Altogether, this can foster sustainable institutional curricular changes that may continue to improve pathology exposure for medical students early on and throughout their education.

It is worth highlighting the philosophical differences in the medical programs studied. Typically, allopathic physicians and MD-granting institutions emphasize understanding pathologies and the therapies available for treatment. Conversely, osteopathic physicians and osteopathic medical schools place a greater emphasis on holistic care and hands-on techniques, along with the study of basic science and disease processes.^30^ A study by George and McCloskey showed that osteopathic students are placed at a relative disadvantage compared to their allopathic peers in terms of exposure and opportunities for advanced education in pathology, such as hands-on microscopy, autopsy demonstrations, and pathology rotations at university-affiliated hospitals.^8^^,^^17^^,^^28^

On the contrary, our study reveals that allopathic students at MSU were generally less satisfied with the amount of pathology instruction in their curriculum, despite traditionally having more opportunities to explore the field. It is also notable that many allopathic students in our study were either unsure of or denied having a “dedicated” pre-clerkship pathology course despite the inclusion of a 1st-year histology/pathology course in the MSUCHM curriculum. This suggests the course may not be conducive for students to appropriately differentiate normal histology from foundational pathologic principle or organ-specific disease processes that constitute a healthy portion of medical school board exam content. Alternatively, some students may simply hold disdain for such content and resort to learning pathology through other means such as 3rd-party resources such as Pathoma or First Aid for the United States Medical Licensing Exam Step 1. As such, these findings warrant further investigation of what UME content students consider to be pathology related, as well as an exploration of modern-day medical school curricula in terms of pathology integration and the causes behind the differences in pathology education and exposure between allopathic and osteopathic medical students.

This study is not without limitations. A notable limitation was the relatively low response rate from each school, especially given the total number of enrolled students, with a significant skew favoring responses from osteopathic students compared to allopathic students. Despite efforts to reach all enrolled students, the large number of enrollees, geographic variability (students at different campuses and clinical rotation sites), and varying administrative staff at each institution may have hindered students' participation. Other factors, including geographic variations across the state of Michigan and differing training stages of the students complicate conclusions about whether the results genuinely reflect differences in opinions among students at each school. Despite these challenges, we believe our findings can guide future studies on furthering this essential topic.

## Conclusions

Our study highlights an overall lack of exposure to pathology among medical students, which likely contributes to a misinformed perception of the field and how it relates to the practice of medicine regardless of their eventual specialty of choice. Improving the quality, delivery, and relevance of pathology content in foundational coursework and clinical applications throughout their education can better inform them about the importance of the field and help them make more informed decisions regarding their future career choices. Differences in the pathology curricula between allopathic and osteopathic schools need further investigation to understand their impact on student perceptions. Future research should focus on examining formative pathology experiences, sources of information about the specialty, and how these shape student interest in pursuing pathology. This study is significant as it sheds light on the limited nature of pathology content in medical schools and aims to inspire further studies to enhance pathology education across the U.S. More research is necessary to assess the quantity and quality of pathology education nationwide, ensuring effective training for all medical students.

## Funding

The article processing fee for this article was funded by an Open Access Award given by the Society of ‘67, which supports the mission of the Association for Academic Pathology to produce the next generation of outstanding investigators and educational scholars in the field of pathology. This award helps to promote the publication of high-quality original scholarship in *Academic Pathology* by authors at an early stage of academic development.

## Declaration of competing interest

The authors declare that they have no known competing financial interests or personal relationships that could have appeared to influence the work reported in this paper.
