# Supplementary material for: Osteopathic versus allopathic medical school pathology curricula: a survey of medical students at Michigan State University
Source: Acad Pathol. 2025 Feb 18;12(1):100164. doi: 10.1016/j.acpath.2025.100164 (PMC11880694; doi:10.1016/j.acpath.2025.100164)
Supplement: Multimedia component 1 [file mmc1.docx]

**Supplemental Material 1**

| **MSU Medical School Pathology Curricula Study CONSENT FORM FOR SURVEY:**  You are being asked to participate in a research study. The purpose of the study is to understand pathology curricula delivery and pathology experience differences among current MSU allopathic and osteopathic students. You will be asked to complete this survey, which takes approximately 3-5 minutes (22 multiple choice questions). You are free to skip any question you do not want to answer. This survey is voluntary and anonymous. You can withdraw at any time before submitting the survey and must be 18 years or older to participate. If you have any questions, please contact Dr. Paul Kowalski at pauljk@msu.edu. By clicking "I agree" below and submitting the survey after its completion, you indicate that you voluntarily agree to participate in this research study.   1. I agree 2. I disagree |
| --- |
| **What medical school do you attend?**   1. MSU College of Osteopathic Medicine (MSU-COM) 2. MSU College of Human Medicine (MSU-CHM)   **What year in medical school are you?**   1. 1st year 2. 2nd year 3. 3rd year 4. 4th year 5. 5th year or greater (PhD dual program)   **Does your school have a dedicated pathology course in pre-clerkship?**   - 1. No   2. Yes   3. Unsure   4. NA   **Does your school offer a required pathology clerkship rotation?**   1. No 2. Yes 3. Unsure 4. NA   **Do you consider pathology more so a surgically- or medically-based specialty?**   1. Equally surgically- and medically-based 2. Medically-based 3. Surgically-based 4. NA   **Have you ever interacted with a pathologist at your medical school or in an extracurricular setting?**   - 1. No   2. Yes   3. Unsure   4. NA   **If “Yes” to the question above, please choose which setting(s) from the following options below (can select more than one option).**   1. Medical School 2. Shadowing 3. Volunteering 4. Previous Job (e.g., Histotechnologist) 5. Prior Degree (e.g., Masters of Laboratory Science) 6. Other   **How many different pathologists have taught you?**   - 1. 0-1   2. 2-3   3. 4-6   4. 7+   5. NA   **What top 3 resources do you most utilize to learn pathology content in your medical curriculum?**   1. Course Lectures 2. Pathology Interest Group 3. Textbooks 4. First Aid 5. Pathoma 6. PathElective.com 7. Social Media (eg., Twitter, Facebook, etc.) 8. Other   **Is pathology regularly integrated during other systems courses throughout your pre-clerkship education?**   - 1. No   2. Yes   3. Unsure   4. NA   **Did you know that pathologists went to medical school?**   - 1. No   2. Yes   3. NA   **Have you observed the clinical work of a pathologist at any point during your medical experience?**   - 1. No   2. Yes   3. Unsure   4. NA   **I am interested in pursuing pathology as a residency.**   - 1. Definitely not   2. Probably not   3. Might or might not   4. Probably yes   5. Definitely yes   6. NA   **How much exposure to pathology have you had at your school?**   - 1. None at all   2. A little   3. A moderate amount   4. A lot   5. A great deal   6. NA   **I believe my formal pathology exposure in medical school should be…**   - 1. Much less   2. Somewhat less   3. About the same   4. Somewhat more   5. Much more   6. NA   **How satisfied are you with the quality of instruction related to pathology at your medical school?**   - 1. Extremely dissatisfied   2. Somewhat dissatisfied   3. Neither satisfied nor dissatisfied   4. Somewhat satisfied   5. Extremely satisfied   6. NA   **What level of importance would you rate the work of a pathologist in patient care in comparison to other specialties (e.g., family medicine, surgery, etc.)**   - 1. Not at all important   2. Slightly important   3. Moderately important   4. Very important   5. Extremely important   6. NA   **Pathology exposure is equivalent to most other biomedical science content (eg.. pharmacology, physiology, anatomy, etc.) at my medical school.**   - 1. Strongly disagree   2. Somewhat disagree   3. Neither agree nor disagree   4. Somewhat agree   5. Strongly agree   6. NA   **My medical school curriculum makes obvious which content being taught is pathology-related.**   - 1. Strongly disagree   2. Somewhat disagree   3. Neither agree nor disagree   4. Somewhat agree   5. Strongly agree   6. NA   **Pathology content is a priority of my medical school curriculum.**   - 1. Strongly disagree   2. Somewhat disagree   3. Neither agree nor disagree   4. Somewhat agree   5. Strongly agree   6. NA   **I have a strong general interest in learning pathology and pathophysiology.**   - 1. Strongly disagree   2. Somewhat disagree   3. Neither agree nor disagree   4. Somewhat agree   5. Strongly agree   6. NA   **How well do you feel your program has prepared you in understanding pathologists’ role in the healthcare team?**   - 1. Not well at all   2. Slightly well   3. Moderately well   4. Very well   5. Extremely well   6. NA   **I am comfortable with pathology questions on board exams.**   - 1. Extremely uncomfortable   2. Somewhat uncomfortable   3. Neither comfortable nor uncomfortable   4. Somewhat comfortable   5. Extremely comfortable   6. NA |
